# The dark side of opioids in pain management: basic science explains clinical observation

**DOI:** 10.1097/PR9.0000000000000570

**Published:** 2016-09-08

**Authors:** Cyril Rivat, Jane Ballantyne

**Affiliations:** a Université de Montpellier, Montpellier, France, Institut des Neurosciences de Montpellier, INSERM U1051, Montpellier, France; b Department of Anesthesiology and Pain Medicine, University of Washington School of Medicine, Seattle, WA, USA

**Keywords:** Opioids, Hyperalgesia, Central sensitization, Pain chronification

## Abstract

**Introduction:**

In the past 2 decades, opioids have been used increasingly for the treatment of persistent pain, and doses have tended to creep up. As basic science elucidates mechanisms of pain and analgesia, the cross talk between central pain and opioid actions becomes clearer.

**Objectives:**

We aimed to examine the published literature on basic science explaining pronociceptive opioid actions, and apply this knowledge to clinical observation.

**Methods:**

We reviewed the existing literature on the pronociceptive actions of opioids, both preclinical and clinical studies.

**Results:**

Basic science provides a rationale for the clinical observation that opioids sometimes increase rather than decrease pain. Central sensitization (hyperalgesia) underlies pain chronification, but can also be produced by high dose and high potency opioids. Many of the same mechanisms account for both central pain and opioid hyperalgesia.

**Conclusion:**

Newly revealed basic mechanisms suggest possible avenues for drug development and new drug therapies that could alter pain sensitization through endogenous and exogenous opioid mechanisms. Recent changes in practice such as the introduction of titration-to-effect for opioids have resulted in higher doses used in the clinic setting than ever seen previously. New basic science knowledge hints that these newer dosing practices may need to be reexamined. When pain worsens in a patient taking opioids, can we be assured that this is not because of the opioids, and can we alter this negative effect of opioids through different dosing strategies or new drug intervention?

## Abstract 2

Although there is no doubt about opioids' ability to relieve pain in the short term, it is not always clear why longer-term analgesic efficacy seems to be impaired. Tolerance and hyperalgesia have been suggested as mechanisms for opioid analgesic deterioration. But could there also be an effect of opioids on pain itself?

The Angelic face of Opium is dazzlingly seductive, but if you look on the other side of it, it will appear altogether a Devil. There is so much poison in this All-healing Medicine that we ought not to be by any means secure or confident in the frequent and familiar use of it.

**Thomas Willis “Medicine in Man's Body” VII i 128 1848**

The Angelic face of Opium is dazzlingly seductive, but if you look on the other side of it, it will appear altogether a Devil. There is so much poison in this All-healing Medicine that we ought not to be by any means secure or confident in the frequent and familiar use of it.

**Thomas Willis “Medicine in Man's Body” VII i 128 1848**

## 1. Introduction

Opioid drugs have been used for the relief of pain for millennia, understanding that these drugs are also highly addictive. To the limits of their availability, opioids were used therapeutically for many centuries with little understanding of why this precious extract from the opium poppy would have such profound effects in humans. But in the 20th century, huge changes occurred both in the clinical use of opioids and in the scientific understanding of why opioids produce both pain and addiction. Parallel events occurred. In part related to much greater availability of opium, its derivatives and synthetics, opioids were administered more widely and in markedly higher doses for the management of pain than ever before. At the same time, mechanisms of pain and analgesia, both opioid related and not, were appreciated at an increasingly microscopic level, culminating in today's understanding of the significant role of endogenous opioid systems in pain and analgesia, and the part played by these systems in the survival and evolutionary development of the species and even in the polymorphisms that arise in individuals.^20^

Let us consider what happened clinically with opioids in the twentieth century. The large swings in clinical opioid prescribing that have occurred over the century because of fears of unnecessary suffering due to underuse, pitched against fears of unfettered addiction due to overuse, have been widely written about and will not be belabored here. What is of greater relevance in the context of the present article is the changes in clinical practice that have led to people being exposed to high doses of opioids given continuously over long periods of time. The reformulation of opioids into long-acting preparations, the idea of giving opioids round the clock, the *titrate-to-effect principle* with open-ended dose escalation, and the *breakthrough pain concept* have all contributed to this. This is in stark contrast to prior usage where opioids were generally provided in what would today be considered low doses, and were much more likely to be given intermittently, as needed, rather than round the clock.

What has emerged from this clinical “experiment” is that higher doses and more prolonged continuous use of opioids increase the risk of adverse effects for individuals, including overdose and death, falls and fractures, road traffic accidents, endocrinopathies, chronic constipation, lack of disease resistance, neonatal abstinence syndrome for offspring, and refractory tolerance when treating acute or end of life pain.^84,100^ It also increases the risk of adverse effects (including death) for society arising largely from addiction on the part both of individuals being prescribed opioids for pain, and those around them who obtain prescription opioids through theft or diversion.^77^ But these now indisputable adverse effects aside, we must ask whether the new principles of opioid prescribing have actually improved analgesia, especially for those seeking relief from chronic persistent pain. We have evidence now that neuroadaptation interferes with opioids' ability to provide long-term analgesia,^2,8,73^ especially when opioids are given continuously and may actually produce opposite effects ie, increase existent pain or facilitate chronic pain development.^3,40,53,96,107^ We have clinical evidence that dose reduction or opioid discontinuation through tapering often improves analgesia.^11,15,47,55,104,112^ We have early evidence that a large proportion of those currently treated for long term with opioids are not meeting treatment goals for either pain relief or function, and that those taking low to moderate doses intermittently gain as much as high dose users with less harm.^27,34,38,106^ These are all clinical indicators that suggest that the new principles of opioid prescribing – use round the clock, titration to effect, and the concept of breakthrough – need to be reconsidered. And what better way to start than with the science behind the adaptations that seem to be interfering with opioids' ability to provide effective long-term analgesia?

## 2. Tolerance and hyperalgesia: neurobiological adaptation to opioid analgesia

The use of potent analgesic opioids such as morphine is motivated by their inhibitory effects on pain transmission. Opioid receptors are members of the G protein-coupled receptor (GPCR) superfamily characterized by the presence of 7 transmembrane regions. Opioid receptors belong to the well-known Gi/o class of GPCRs. It is commonly accepted that the main inhibitory effects of opioid on pain transmission are due to the stimulation of μ-opioid receptor (MOP) resulting in an inhibition of adenylyl cyclase and ion channels. When activated, MOP produces hyperpolarization of neurons decreasing the transmission of nociceptive information through the activation of components of the mitogen activated proteins (MAP) kinase cascade.^116^

Tolerance is characterized by a progressive lack of response to morphine that can be overcome by increasing the dose, whereas hyperalgesia is a sensitization process by which opioids, paradoxically, cause pain hypersensitivity.^56^ Both mechanisms lead to the decreased efficacy of opioid analgesic effects. These adaptive phenomena have been studied for decades with notable insights gained from experiments, usually pharmacological and may be explained by 2 different biological processes.^57^ The *within-system adaptation process* suggests that drug administration elicits an opposing reaction within the same system in which the drug elicits its primary action. Such an adaptive response acts to progressively neutralize the drug's effect and is exemplified by mechanisms of opioid receptors desensitization (see below) and is referred to as tolerance. The other conceptual advance on decreased analgesic effects of opioids is the relationship between the paradoxical pain hypersensitivity produced by acute or chronic opioid administration and the development of resistance to analgesic effects. This has given rise to the concept of *between-system adaptations*. To illustrate this point, it has been reported in both laboratory and clinical studies that repeated or acute opioid administration induces not only analgesia, but also hyperalgesia.^2,93^ The development of hyperalgesia has been evidenced after potent short-acting MOP agonist such fentanyl,^23^ remifentanil,^36^ buprenorphine,^113^ but also after potent long-acting MOP agonist such as morphine.^66,68,109^ Interestingly the milder MOR agonist tramadol has also been shown to produce pain hypersensitivity,^61^ suggesting that the adaptive response manifesting as hyperalgesia seems to be a feature common to differing MOP agonists. In animal studies, opioid-induced pain hypersensitivity can be observed after high-doses and/or chronic opioid administration but also after ultra-low doses (around 1–10 μg/kg in vivo) revealing the excitatory properties of opioid receptor.^30,31,41,97^ This hyperalgesia is mediated by the activation of specific pronociceptive processes^23,39,49,68,78,108,109^ which also lead to an exaggeration of injury-induced hyperalgesia.^17,43,62,87,89,90,92^ Interestingly, experimental studies report that the prevention of opioid-induced pain hypersensitivity is able to restore opioid analgesic effects.^87,108,119^ Thus these observations strongly support the fact that decreased analgesic effect is not due only to an alteration at the opioid receptor level (tolerance) but could also be associated with an activation of pronociceptive systems triggered by the opioid that counteracts opioid analgesic effects.^2,45,78,98^ As proposed by Célèrier et al.^21^ in 1999, the net effects of opioids such as morphine may be due in reality to the balance of the predominant activation of pain inhibitory systems but these effects would be partially unmasked by the concomitant activation of pain excitatory systems. Many features of opioid-induced hyperalgesia can be viewed in terms of adaptive response intended to normalize net activity by engaging opposing or compensatory regulatory mechanisms or signaling pathways to reduce opioid responses. This phenomenon refers to the opponent process theory.^98,99^ Considering the multiple cellular events involved in the adaptive response produced by opioid exposure, it is important to note that most of them are also common to those involved in the development and maintenance of chronic pain. This suggests that pain vulnerability may be facilitated in patients taking opioid medication and that unfortunately opioid-induced pain vulnerability may persist even after the opioid prescription has stopped. That means that opioid may participate in the facilitation of the transition from acute to chronic pain.

### 2.1. Mechanisms of tolerance and hyperalgesia induced by opioids

As noted above, different concepts have been proposed to explain the development to tolerance. First, with regards to the *within-system adaptation process*, changes in numbers of receptors, signaling proteins, and levels of opioid receptor phosphorylation are part of the alterations that reflect cellular adaptive changes to opioid exposure. The MOP (and δ Opioid receptor [DOP]) activation initiates a cascade of events (phosphorylation, receptor endocytosis, intracellular sorting, and recycling) leading to desensitization and resensitization which are important regulatory processes that control signaling and cellular response.^51^ In this process, receptor trafficking is an important aspect of opioid regulation through the involvement of dynamin and Beta-arrestin. Opioid receptor desensitization can occur through phosphorylation by G-protein couple receptor kinase and subsequent arrestin binding.^5^ Phosphorylation of specific residues through increased activation of ERK1/2 (extracellular signal-regulated kinases), G-protein couple receptor kinases in the intracellular domains of MOP is widely accepted to precede and perhaps cause desensitization. This phenomenon has been described in a recent review and will not be discussed here. In addition, desensitization of opioid receptors occurs after interaction with other GPCRs called heterodimerization^46,54,80^ or when a ligand binds to a specific GPCR, causing the inactivation/desensitization of a different, unrelated, and unstimulated GPCR through intracellular signaling. This process is called heterologous desensitization.^14,28,58,102,103^

**Figure 1.**
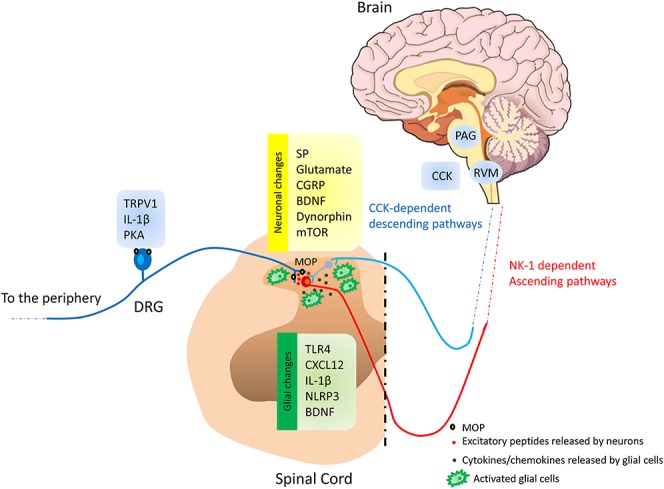
General view of opioid-induced pain sensitization. Peripheral changes may occur in the primary afferent neurons through enhanced expression in transient receptor potential vanilloid 1 and activity of the protein kinase-A (PKA) and up-regulation of IL-1β in satellite cells that produce increased release, in the dorsal horn of the spinal cord, of excitatory peptides such substance P (SP) and calcitonin gene-related peptide (CGRP) and of glutamate. In addition, at the spinal cord, a complex interplay between neurons and glial cells may occur. Neurons are sensitized by mammalian target of rapamycin (mTOR)-dependent mechanisms after opioid administration. Activated glial cells through direct (TLR4) and/or indirect action of opioid may produce the release of chemokines, cytokines, and *Brain-derived neurotrophic factor* (BDNF) that sensitize neurons leading to overactivity of ascending pain pathways. Activation of descending facilitatory pain pathways after opioid administration through the increase in cholecystokinin (CCK) into the rostral ventromedial medulla (RVM), facilitate the release of excitatory peptide in the spinal cord contributing to the maintenance of long-lasting pain sensitization after short-term opioid exposure.

With regard to the mechanisms involved in opioid-induced hyperalgesia ie, *between system adaptation*, several comprehensive reviews have summarized the cellular and molecular changes that support opioid effects.^2,78,93,98^ Recent important new findings provide additional evidence of opioid-induced pain sensitization. N-methyl-d-aspartate (NMDA) receptor has long been implicated in the development of both opioid analgesic tolerance and opioid-induced hyperalgesia,^21,60,68,105^ especially through potentiation of presynaptic NMDA receptor.^120^ The importance of NMDA neurotransmission in opioid-induced tolerance and hyperalgesia has recently been emphasized by new observations. A whole-genome haplotype-based computational genetic mapping designed to identify genes involved in opioid-induced hyperalgesia was used.^35^ *Mpdz* gene was identified as a candidate to explain differences among inbred mouse strains in developing pain hypersensitivity after short-term morphine exposure. The *Mpdz* gene and the associated Multi-PDZ domain protein 1 (MUPP1) have been shown to be involved in NMDA-dependent synaptic functions. Specifically, Multi-PDZ domain protein 1 associated with Ca^2+^/calmodulin-dependent protein kinase II and SynGAP constitutes a synaptic complex that regulates p38 MAP kinase activity and NMDA receptor-dependent synaptic α-amino-3-hydroxy-5-methyl-4-isoxazolepropionic acid receptor potentiation.^59^ These data support previous reports showing that the MOP agonist, remifentanil produces long-term potentiation in dorsal spinal cord after cessation of opioid administration.^36^ Altogether, this suggests that opioids may facilitate “spinal pain memory” and may facilitate long-lasting changes in pain processing pathways. As well as the involvement of NMDA receptors in opioid-induced hyperalgesia, spinal MAP kinase signaling pathway, including extracellular signal-regulated kinase^19,115^ and calmodulin-dependent protein kinase II^26^ have also been implicated in mediating this phenomenon. MAP kinase has been shown to be responsible for transient receptor potential vanilloid 1 over-expression in sciatic nerve, dorsal root ganglia, and spinal cord after repeated morphine administration.^25,110^ The blockade of transient receptor potential vanilloid 1 or MAP kinase activation reduces analgesic tolerance and thermal pain hypersensitivity. Additional signaling pathways have been implicated in opioid-induced hyperalgesia. Recently, the mammalian target of rapamycin (mTOR), a serine-threonine protein kinase mTOR has been shown to be increased in dorsal horn neurons after repeated intrathecal administrations of morphine. Mammalian target of rapamycin contributes to the development of both morphine tolerance and hyperalgesia through phosphoinositide 3-kinase/Akt pathway in dorsal horn neurons since blockade of mTOR reduces those phenomena.^119^ Thus the spinal cord appears central to various mechanisms and signaling pathways supporting pain hypersensitivity observed after chronic opioid administration (Fig. 1). However, the development of paradoxical pain hypersensitivity after opioid administration has also been related to activation of the descending nociceptive pathways originating from the rostral ventromedial medulla^109^ through enhanced endogenous cholecystokinin (CCK) activity^118^ which, in turn, may promote the upregulation of spinal dynorphin and enhances primary afferent neurotransmitter release (such as calcitonin gene-related peptide^42^; Fig. 1). Interestingly, spinal dynorphin has been shown to be regulated by epigenetic mechanisms during morphine exposure.^64,65^ Indeed, it has been shown that escalating doses of subcutaneous morphine induce enhanced expression of aceH3K9 in dorsal spinal cord^64^ regulating the expression of *Pro-dynorphin* and *BDNF (Brain-derived neurotrophic factor)*, as well. Moreover, repeated intraperitoneal Histone acetyltransferase inhibitor injections reduced both morphine tolerance and pain hypersensitivity, whereas histone deacetylase inhibitor injection produced the opposite effects ie, prolongation of morphine hyperalgesia and tolerance. Whether epigenetic alterations are induced after acute opioid administration remains to be determined, especially in the context of tissue injury, but these data strongly support long-term neuronal changes caused by opioid exposure.

### 2.2. Opioid-induced neuroinflammatory-like state

Abundant evidence suggests that opioid can also produce neuroinflammatory responses in both peripheral and central nervous systems. Microglia-to-neuron signaling is known to play a key role in opioid-induced tolerance and hyperalgesia. The implication of microglial cells in pain sensitization is quite complex. Glia cells have been shown to contribute to opioid-induced hyperalgesia through the release of proinflammatory cytokines and chemokines.^72,81,82,117^ To support the inflammatory process activated after opioid administration, administration of glial metabolic inhibitors, receptor antagonists or cytokine inhibitors, attenuate the development of morphine tolerance. For instance, interleukin-1 (IL-1)β is increased after chronic intrathecal morphine administration and the blockade of spinal IL-1β receptor is effective in reducing both the development of tolerance to morphine analgesia and hyperalgesia and allodynia observed after repeated morphine administration.^52^ At the cellular level, acute as well as chronic morphine administration produces enhanced protein expression of IL-1β from satellite cells in the dorsal root ganglia through MMP9 activation.^12,76^ Further investigations have recently been conducted to elucidate the role of opioid-induced glial activation in the modulation of opioid effects. Microglia through P2X4 stimulation has been more specifically involved in morphine-induced hyperalgesia^39^ rather than tolerance suggesting distinct mechanisms between these 2 phenomena. Although, tolerance and hyperalgesia may share some common mechanisms,^56,86^ additional investigations should be conducted in the future to confirm the data obtained by Ferrini et al. In addition, it has been shown that the microglia-specific subtype of Ca^2+^-activated K+ (BK) channel is responsible for generation of morphine-induced hyperalgesia and tolerance to its analgesic effect.^44^ Of note, opposition of analgesia by proinflammatory cytokines is rapid, occurring 65 minutes after intrathecal opioid administration suggesting nonclassic opioid actions on the production of proinflammatory cytokine.^52^ Actually, opioid ligands such as morphine have been shown to directly bind the microglial activate marker, innate immune receptor toll like receptor 4 (TLR4) resulting in decreased morphine antinoception and hyperalgesia. Such effects have been reported in both the spinal cord^50^ and the periaqueductal gray (PAG).^37^ Stimulation of TLR4 by opioid activates ceramide metabolic pathway in spinal glial cells. That would lead to the activation of the sphingosine 1 phosphate kinase responsible of enhanced production of TNFα, IL1-β and IL-6^75^ and may represent one of the mechanisms by which opioids induce a rapid cytokine overexpression. One of the most prominently reported cascades activated by opioid exposure is the MAP kinase pathway which a collection of serine/threonine-specific protein kinases. Three key kinases of this response system are p38, c-Jun N-terminal kinase (JNK), and extracellular signal-regulated kinase, the phosphorylation of which results in an active functional signaling complex. For instance, morphine produces phosphorylation of p38 within microglia.^32,48,115^ Altogether, the contribution of proinflammatory cytokines in pain hypersensitivity suggests a participation of these molecules as antiopioid system that counteract opioid analgesia. These observations point out the implication of cytokines as heterologous system in the modulation of opioid analgesia, in agreement with the *within system adaptation process* theory. Of note, spinal astrocytes have also been implicated in the development of opioid-induced hyperalgesia^13,97^ through the involvement of the c-Jun N-terminal kinase pathway and IL-1β. Altogether this observation strongly provides favor for gliosis activation after short-term opioid exposure.

Recent advances demonstrate the importance of chemokines in the modulation of opioid analgesic effects. Chemokines constitute a family of small secreted proteins which were initially described as chemoattractive molecules for lymphocytes.^71^ However, in addition to their classical role in the immune/inflammatory reaction, some chemokines are also detected in resting conditions by neurons and glial cells producing a glio- and neuromodulatory activities.^18,94^ Chemokines are also modulated by morphine exposure within the central nervous system. As the cytokines, chemokines are known to play a critical role in functional adaptation after opioid exposure. This statement emerges from different observations. Works by Watkins' team firstly demonstrated that the chemokine CX3CR1/fractalkine oppose morphine analgesia.^52^ CX3CR1/fractalkine-induced modulation of morphine analgesia takes place also in the PAG.^24^ One of these chemokines, stromal derived factor 1 (SDF-1), also called CXCL12 is a member of the CXC family of chemokines and it binds to 2 receptors: the G-protein coupled transmembrane (CXC motif) receptor 4 (CXCR4), (also identified as a coreceptor for the T cell tropic human immunodeficiency virus-1) and the CXCR7 receptor.^6^ Heterologous desensitization of MOP and DOP has been documented after stimulation of CXCR4.^81,82,101^ For instance, CXCR4 receptor activation in the PAG, by prior local treatment with CXCL12 markedly reduced the antinociceptive effects of opioid agonists injected in the same site.^101^ The CXCL12/CXCR4 system has also been implicated in chronic morphine administration-induced tactile hypersensitivity.^117^ The participation of spinal CXCL12/CXCR4 system in morphine tolerance has also been evidenced. Intrathecal injection of CXCL12 reduced morphine analgesic effects.^91^ Altogether, this suggests that the spinal chemokine CXCL12 may contribute to the development of opioid tolerance through *within* and *between* adaptations.^72^

## 3. Pain chronicization produced by opioid: concept of latent pain sensitization

### 3.1. Unmasking opioid-induced persistent pain sensitization

**Figure 2.**
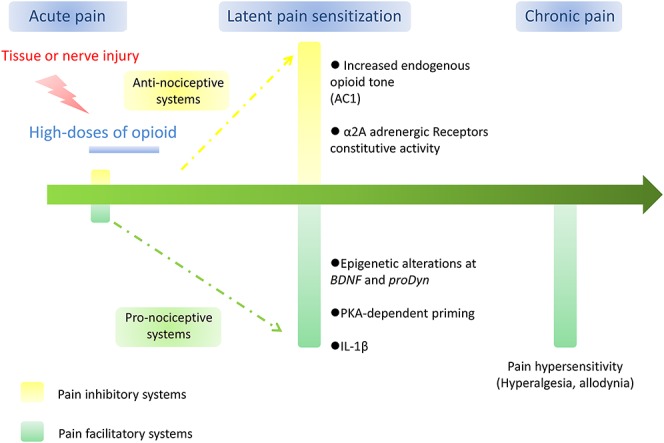
Hypothesis on the transition from acute to chronic facilitate by opioid administration. Acute tissue or nerve injury produces an increased activity in pain facilitatory systems that can be exaggerated by treatment with high doses of opioid. After remission and cessation of opioid administration, increased endogenous pain inhibition through opioid and α2A adrenergic receptors constitutive activity suppress sustained hyperalgesia that may depend on epigenetic alterations at *BDNF* and *proDyn gene* expression, protein kinase-A in primary afferent neurons and increased expression of the pro-inflammatory cytokines IL-1β. This results in the development of latent pain sensitization that may be associated with long-term pain vulnerability that could facilitate the development of chronic pain.

Several recent bodies of evidence suggest that opioid exposure can induce relatively long-term activation of pronociceptive systems leading to pain chronicization. First, it has been observed that after hindpaw inflammation, animals develop an enhanced hyperalgesic response to a second induction of inflammation 7 days later.^89,90^ This enhanced response is exaggerated when fentanyl is administered during the first episode of tissue inflammation suggesting that both painful inflammation and opioids can facilitate pain sensitization, making animals vulnerable to future pain for at least one week. Similar effects were observed in an animal model of postoperative pain.^16^ Second, in a different, but very interesting, experimental paradigm, nociceptive hypersensitivity progressively disappeared over 12 days after cessation of repeated opioid administration.^22^ However, administration of naloxone induced a dramatic decrease in the nociceptive threshold (precipitated hyperalgesia) when administered 8 weeks after opioid administration. Importantly, naloxone-precipitated hyperalgesia was not observed in opioid-naive rats. This suggests that after opioid exposure, animals develop an activation of pronociceptive systems that persists for at least 8 weeks after opioid exposure although this enhanced pronociceptive becomes masked by endogenous opioid-mediated antinociceptive systems in just few days. Hyperalgesia may then be evident when these endogenous opioid-mediated antinociceptive systems are inhibited by naloxone. Taken together all these results suggest that the resolution of opioid-induced hyperalgesia is not due to a rapid extinction of pronociceptive systems, leading to a return to normal nociceptive responding (homeostatic state) but is rather due to a counter-adaptation by inhibitory systems dependent on endogenous opioid release.^22^ This phenomenon leads to the establishment of a new state, which has been referred to as allostasis (meaning “maintaining stability [or homeostasis] through change”^69,70^ and which depends on a high-level balance between these 2 opposing nociceptive-related systems, Fig. 2). It has been proposed that this allostatic state may be a state of pain vulnerability. To illustrate this state of pain vulnerability, it has been shown that the administration of a small dose of heroin (0.2 mg/kg), ineffective in inducing hyperalgesia in normal rats, triggered substantial long-lasting hyperalgesia when given several days after prior opioid exposure.^22^ Similar effects were obtained when spinal applications of low dose BDNF or dynorphin were applied after opioid-induced hyperalgesia resolved^65^ suggesting that opioids produce a general state of pain sensitization. Moreover, stress, which is well known to induce analgesia in normal rats^1,79^ produces hyperalgesia in rats previously treated with opioids.^88^ Since such stress-induced hyperalgesia was observed in animals that were returned to basal pain sensitivity; this phenomenon has been named “latent pain sensitization.” Importantly, such a phenomenon can be observed up to 119 days after opioid/injury exposure.^88^ Opioid-induced long-term pain vulnerability may increase pain response after various tissue injuries such as pancreatic inflammation,^63^ hindpaw inflammation,^60^ incision,^16,62^ or nerve injury.^67^ Importantly, the development of latent pain sensitization has recently been reported in a preliminary human volunteer study. The administration of 2 mg/kg naloxone produced a decreased pain threshold 7 days after a mild heat injury as revealed by heat pain threshold and secondary hyperalgesia.^83^ Naloxone response seems to be heterogeneous between human volunteers suggesting interindividual variation in the development of latent pain sensitization. To challenge this observation, this clinical paradigm should be further tested. It would be of interest to identify markers of vulnerability to chronic pain to prevent the transition from acute to chronic pain, especially in the context of postoperative pain. Altogether, these observations suggest that opioid exposure may sensitize individuals to subsequent different painful and nonpainful stimuli making opioid a critical factor in the transition from acute to chronic pain as evidenced after surgery.^96,107^ This observation raised important questions about the neurobiological support of opioid-induced long-lasting neuroplasticity.

### 3.2. Long-term adaptive changes induced by opioid exposure

With regard to the mechanisms supporting opioid-induced long-term pain sensitization, some reports have recently been published and deserve some attention. First, it seems that opioid-induced pronociceptive activity may persist long after cessation of opioid administration. For instance, Loram et al.^67^ showed that 2 days after cessation of opioid exposure, enhancement of IL-1β mRNA can be observed in the spinal cord and trigeminal nucleus caudalis suggesting that sustained inflammatory mechanisms may be responsible for opioid-induced pain chronicization. This observation has to be related to a recent report showing that short-term opioid exposure (5 days) prolongs nerve injury-induced pain hypersensitivity for several weeks.^43^ This observation is consistent with the idea that opioid produces long-term alterations in pain sensitization process that facilitates the initiation and/or the maintenance of chronic pain state. The main mechanism supporting such a phenomenon seems to be independent of opioid receptor, involving the NOD-like receptor protein 3 inflammasome, a protein complex that activates toll Like Receptor 4 (TLR4), P2X7 receptor, caspase-1, or IL-1β in dorsal spinal microglial.^43^ Actually, these data support the critical role of microglia not only in opioid-induced hyperalgesia and tolerance but also in long-term pain sensitization observed after brief exposure to opioid.

Other mechanisms have been proposed to explain opioid-induced persistent pain sensitization. As mentioned above, opioids may trigger epigenetic mechanisms that produce hyperalgesia and tolerance. Since epigenetics include processes that control long-term gene expression, one may speculate that opioid-induced latent pain sensitization may be supported by histone modifications, DNA methylation and/or miRNA synthesis. Along these lines, it has been reported that histone acetylation may drive exaggerated incision-induced pain hypersensitivity after morphine exposure through changes in *BDNF* and *pro-dynorphin* gene expression in the spinal cord.^95^ These epigenetic mechanisms may have particular interest to partially explain the latent pain sensitization produced by opioid through long lasting alterations in neuronal functioning after opioid exposure. Finally, repeated stimulations of MOP produce long-term sensitization of nociceptors through specific mechanisms different to those produced by inflammatory stimulus.^4^ Indeed, specific signaling pathways involving protein kinase-A has been shown to support long-lasting hyperalgesia after prostaglandin E2 (PGE2) injection in DAMGO-pretreated animals. This report demonstrates a novel form of hyperalgesic priming that may facilitate the transition from acute to chronic pain. This suggests that opioid may act also at primary afferent nociceptors inducing a priming effect that sensitizes animals to subsequent painful stimulus. Finally, the concept of latent of pain sensitization has also been investigated after inflammatory pain. It has been recently shown that adenylate cyclase 1 and MOP, DOP, kappa-opioid receptor, and α2A adrenergic Receptors constitutive activity are implicated in the sustained suppression of pain hypersensitivity after acute inflammation.^29,114^ The question remains regarding the specific implication of those cellular mechanisms in latent pain sensitization induced by opioid exposure. A better characterization of the neurobiological markers of opioid-induced latent pain sensitization should be considered in the future to improve the use of opioids for the management of pain including limiting the risk of pain chronification (Fig. 2).

## 4. Clinical implications of opioid-induced neuroadaptations

The changes in opioid prescribing that occurred over the past few decades occurred not because of our greater understanding of basic mechanisms of pain and analgesia, but despite it. The changes in opioid prescribing began in the 1980s when palliative care specialists approached the pharmaceutical industry and asked them to reformulate morphine to become long-acting so that cancer patients would have better pain control with fewer peaks and troughs in their analgesic levels. The reformulation of several other opioids rapidly followed. Palliative care specialists also promoted the idea that opioids should be given at regular intervals rather than on demand, escalating dose with the aim of relieving as much pain as possible.^111^ The *titrate-to-effect* principle whereby opioid doses would be increased to counteract not only increases in pain, but also increases in tolerance, was born. One more change is relevant, and that is the introduction of the concept of *breakthrough pain*.^74,84^ When it became clear that there is no ideal dose of long-acting opioid that covers all eventualities and compensates for the changes in pain level that occur with changes in emotional and physical factors, it was proposed that short-acting opioids should be given in addition to long-acting, and given when pain emerges through the base analgesic regimen. Before the advent of long-acting opioids, there was no need for a breakthrough pain concept, because the standard way of prescribing was *as needed*. The principles promoted by palliative care specialists were soon extended to the treatment of chronic pain. Suddenly a much wider population was being treated with opioids and for much longer. And in line with the palliative care specialists' advancement of the *titrate-to-effect principle* and the *breakthrough pain concept*, doses used were higher than ever previously seen.^7,10,100^

Although mechanisms of opioid tolerance and hyperalgesia had begun to be elucidated in the laboratory as early as the 1980s, clinicians did not know quite what to make of it. After all, opioids had been successfully used for the treatment of pain for millennia: it must be that opioids' antinociceptive effects generally override their pronociceptive effects, and that tolerance can be overcome by dose escalation. And initially, the *use round-the-clock, titrate-to-effect*, and *breakthrough pain concepts* seemed to be the answer. That is until the 1990s, when in the clinical space, we began to see that under some circumstances, opioids seemed to be making pain worse rather than better.^9^ This was particularly true when opioids were used either at high doses or with high potency. Lured by the fact that each dose escalation seemed capable of restoring analgesia, dosing went up and up, and the fear that such dosing might actually be worsening pain, or producing adaptations that compromise opioids' ability to provide pain relief, went largely unheeded.

Recent progress in the laboratory has brought us to the point where the pronociceptive effects of opioids and their clinical importance cannot be denied. These can no longer be seen as changes that will reverse as soon as opioid treatment is stopped: animal and some human studies suggest that many of the changes have prolonged effects. Common mechanisms contribute to opioid induced hyperalgesia and pain chronification, such as NMDA receptor activation,^33,53,85^ meaning that it is not always possible to determine whether an increase in pain should be attributed to pain itself or could result from continued administration of opioid. Or indeed, whether discontinuation of opioid therapy will improve pain or affect any sort of rescue in terms of reversing the changes. Whether through cellular processes such as receptor trafficking, intracellular signaling, NMDA neurotransmission or epigenetic changes, *opioid-induced neuroinflammation* or *latent pain sensitization,* opioid-induced tolerance and hyperalgesia must be seen as potentially irreversible phenomena that should force a reexamination of current opioid dosing practices. At the very least, we know that the newer prescribing tenets have tended to drive higher dose usage, which the science now reveals changes in pain processing that can compromise opioids' ability to provide sustained pain relief, and may even worsen underlying pain and/or facilitate the development of chronic pain.

The new understanding of opioid mechanisms afforded by basic science could lead to the development of novel drug therapies that by targeting epigenetic changes, cellular processes, neuroinflammation and other opioid-induced adaptations, could help reduce pain chronification, reduce tolerance to opioid analgesics, or both. Well-designed clinical studies will be needed to clearly define opioid-induced hyperalgesia and tolerance in humans, and to fully understand the role of dose and dosing strategies in producing the undesirable neuroadaptations that interfere with opioids' ability to offer sustained analgesia. However, what the basic science already does is force us to question whether newer practices such as continuous use and open ended dose escalation, could sometimes be making pain worse rather than better.

## Conflict of interest statement

The authors have no conflicts of interest to declare.
